# Cardio-sarcopenia: A syndrome of concern in aging

**DOI:** 10.3389/fmed.2022.1027466

**Published:** 2022-10-25

**Authors:** De Rong Loh, Ru-San Tan, Wee Shiong Lim, Angela S. Koh

**Affiliations:** ^1^Duke-National University of Singapore (NUS) Medical School, Singapore, Singapore; ^2^National Heart Centre Singapore, Singapore, Singapore; ^3^Department of Geriatric Medicine, Institute of Geriatrics and Active Ageing, Tan Tock Seng Hospital, Singapore, Singapore; ^4^Lee Kong Chian School of Medicine, Nanyang Technological University, Singapore, Singapore

**Keywords:** aging, cardiovascular disease, myocardium, sarcopenia, frailty, prevention

## Abstract

Cardiac alterations in structure and function, namely, the left ventricle, have been intensely studied for decades, in association with aging. In recent times, there has been keen interest in describing myocardial changes that accompany skeletal muscle changes in older adults. Initially described as a cardio-sarcopenia syndrome where alterations in myocardial structure were observed particularly among older adults with skeletal muscle sarcopenia, investigations into this syndrome have spurred a fresh level of interest in the cardiac-skeletal muscle axis. The purpose of this perspective is to summarize the background for this “syndrome of concern,” review the body of work generated by various human aging cohorts, and to explore future directions and opportunities for understanding this syndrome.

## Introduction

The traditional view of cardiovascular aging is that of age-related adaptations in the heart characterized by increased left ventricular (LV) mass (LVM) and LV hypertrophy (LVH), which are often secondary to increased systolic blood pressure mainly mediated by arterial stiffening (1, 2). These changes accumulate throughout the lifetime of an individual, increasing the risk of developing cardiovascular disease (CVD), such as heart failure (HF) and coronary artery disease (2). The incidence of CVD increases with age, rising from ∼78% among adults aged 60–79 years to ∼90% in those aged above 80 years (3). CVD is the leading cause of disease burden in the world, with global prevalence doubling from 271 million to 523 million between 1990 and 2019 (4). Incident CVD mortality increased from 12.1 million to 18.6 million in the same period (4), and accounted for 32% of all deaths. With rapidly aging national populations, these numbers are expected to increase. Despite the known association between cardiovascular aging and CVD, this knowledge has not translated into actionable changes that can specifically target aging-related CVD (2). This underscores the urgent need for in-depth study into the pathophysiology of cardiovascular aging and its prevention, and also highlights the unmet need for specific markers of cardiovascular aging that is “modifiable.” Emerging interests surrounding and research into a novel entity of “cardio-sarcopenia” have provided an added dimension to mainstream understanding of cardiovascular aging, and by extension, opened up new avenues for interventional strategies. This term was coined based on observations of associations between LVM and skeletal muscle mass and function that were independent of systemic risk factors such as hypertension and diabetes mellitus, as well as smaller left heart sizes in older adults with skeletal muscle sarcopenia (5). Observed among community older adults without clinical CVD, these distinctive patterns are hypothesis-generating for a possible syndrome of cardiac aging (5). Data from other cohorts have emerged that support relationships between markers of LV and skeletal muscle structure and function across the sarcopenia spectrum (6, 7).

## Cardio-sarcopenia: A possible upstream marker of the heart failure spectrum

Skeletal muscle sarcopenia occurs with aging but may be accelerated in heart failure states (8). In advanced stages of heart failure, skeletal muscle wasting accompanied by severe exercise intolerance have long been observed in various cohorts (9–11). Several systemic and humoral mechanisms have been invoked as biological interactions (i.e., cross-talk) between the skeletal muscle system and the heart (12–15).

To date, observations pertaining to the cardiac muscle-skeletal muscle axis among non-heart failure cohorts have provided useful insights (Table 1). In a population-based cohort of older Asian subjects without clinical cardiovascular disease, skeletal muscle mass was associated with left ventricular mass, independent of age, diabetes mellitus status, and body size (5). In a selected cohort of frail sarcopenic older European subjects without severe cardiovascular disease (some had mild cardiovascular disease), appendicular lean mass was strongly associated with LVM and cardiac output (6). Although advanced age was associated with loss of skeletal muscle mass, the relationship between LVM and skeletal muscle mass appears to be independent of age (5, 7). Among 228 community adults aged 65–91 years, individuals with low skeletal muscle mass had lower LVM than those without low skeletal mass, without significant interaction between age and LVM (7). These observations are hypothesis-generating for possible *age-related yet age-independent* processes that mediate the cardiac and skeletal muscle systems in older persons.

**TABLE 1:** Summary selection of community cohorts on skeletal muscle and cardiac function/muscle.

Cohort studies, publication year	Study population	Results	Inferences	Details/limitations
Keng et al. (5)	CAS study *N* = 378 Mean age 72 ± 4.4 years No self-reported history of physician diagnosed CVD, stroke or cancer.	Skeletal muscle mass was associated with LV diameter, LVM. Participants with sarcopenia had smaller LV size and lower LVM. LVM was linearly associated with handgrip strength.	Sarcopenic patients had smaller LV and LA sizes with gross preservations in LV function, as well as reduced general skeletal mass and handgrip strength. This suggests a possible syndrome of cardio-sarcopenia.	Cross-sectional study; Asian ethnicity; low risk community cohort.
Pelà et al. (6)	Ancillary study of the Sarcopenia and Physical Frailty In Older People: Multicomponent Treatment Strategies (SPRINT-T) project *N* = 100 selected subjects enrolled from Parma site. Mean age 79 ± 5 years	Appendicular lean mass was strongly and positively correlated with LVM, as well as cardiac output.	Confirmed the concept of cardio-sarcopenia syndrome in a European population of older individuals.	Comorbidities such as heart disease and chronic conditions may account for at least 40% of the study sample. Smaller sample size; cross-sectional study; cohort focused on frail older adults.
Pelà et al. (20)	As above. Additional analyses for blood pressure, physical activity, and medications, etc.	60% showed LVH (assessed by LVM/BSA) with a tendency toward concentric geometry, as assessed by RWT. The main determinants of LVM were BSA and SBP, while RWT was primary correlated with age.	SBP as the main determinant of LVM highlights a key role that hemodynamic condition plays in determining LV geometry in an older population of sarcopenic and physically frail patients. Levels of physical activity did not influence LVM or RWT.	Smaller sample size; cross-sectional study; Ambulatory blood pressure was obtained in only 52% of participants.
Tinti et al. (7)	*N* = 288 Free living individuals Mean age 75.61 ± 6.28 years 50% of the participants had diabetes.	Individuals with low SMM had lower LVM. LVM was significantly correlated with fat and lean mass, as well as with SMM and bone mass.	Confirmed that individuals with low skeletal muscle mass had lower LVM than those without low SMM, as found by Keng et al. (5) Ventricular mass in older adults was significantly correlated with fat and skeletal muscle and bone mass, which persisted even after adjusting for body mass index. Diabetes mellitus exerted a negative interacting influence on the association between age and ventricular mass.	Relatively high proportion of diabetes mellitus in the sample. Prevalence of CVD in the sample was not reported.
Ko et al. (89)	*N* = 67,106 Mean age 40.6 ± 8.1 years Participants with heart conditions, such as CVD, systolic heart failure, hypertrophic, or dilated cardiomyopathy, were excluded.	In 67,106 participants, 19,232 subjects (28.7%) and 1,553 subjects (2.3%) had LVDD and LVH, respectively. SMI was positively associated with E/A ratio and septal E’, whereas E/E’ ratio and LV mass index were negatively associated with SMI. Lower SMI was associated with increased presence of LVDD.	Suggests a role of skeletal muscle mass in the pathogenesis of LVDD (but not LVM).	Studied young and middle-aged adults (not older adults). High prevalence of obesity in LVDD group.

Limited to original articles that have directly imaged skeletal and cardiac organ systems in older adults. CVD, cardiovascular disease; LA, left atrial; LV, left ventricular; LVM, LV mass; LVH, LV hypertrophy; LVDD, LV diastolic dysfunction; BSA, body surface area; RWT, relative wall thickness; SBP, systolic blood pressure; SMM, skeletal muscle mass; SMI, skeletal muscle mass index.

## Cardiac muscle: So, is it big or not big with aging?

The observations seem to run counter to the dogma of aging-associated LVH, especially in the context of hypertension which dominates aging. Traditionally, cardiac aging has been associated with increased LV wall thickness, with or without myocyte hypertrophy (16, 17). High LVM, and not low LVM, has been deemed to be clinically unfavorable (18). Among 3,220 subjects enrolled in the Framingham Heart Study who were 40 years of age or older and free of CVD who were followed up over 4 years, increments in LVM (corrected for height) predicted higher incidence of clinical events, including death, attributable to CVD (18). Interestingly, an earlier report from Framingham had also observed associations between subscapular skinfold thickness (and body mass index, among other variables) and LVM, in multivariable analyses, which may suggest that markers of lean body mass are indeed correlated with LVM (19). These historical considerations appropriately place most of the clinical emphasis on the significance of a large LVM.

In contrast, data from sarcopenic subjects suggest that the spotlight on “LVM” could be widened to include the “lower” end of the LVM spectrum. The observed correlates between LVM and specific measurements of skeletal muscle mass/function, emphasize a need to consider the skeletal muscle system as a possible variable in the evaluation of LVM, in addition to body surface area which is routinely calculated in indexed LVM measurements. This may be particularly important in clinical studies where older adults are the focus. Among older adults, there may be interactions between skeletal muscle sarcopenia and LVM. Aging appears to be associated with lower LVM among sarcopenic subjects (5, 7), suggesting that low LVM may be a phenotype of concern in older adults. Future longitudinal studies are needed to observe how subjects with low LVM evolve over time. However, concentric remodeling and LVH appear to occur when higher levels of physical frailty and sarcopenia are reached, albeit cross-sectionally (20). More understanding of events that occur between low LVM at lower levels of sarcopenia and LVH at higher levels of sarcopenia, would be critical. It is plausible that in the presence of sarcopenia, physiological adaptative responses are exceeded such that the development of LVH becomes pathologically significant. This hypothesis would have implications for the intensity and urgency of skeletal muscle management, in addition to routine hypertension management, in sarcopenic older adults.

The mechanism through which low LVM occurs in the setting of sarcopenia is unknown but potentially intriguing. Studies from autopsy specimens reveal that aging is associated with progressive attrition of myocyte numbers, in addition to hypertrophy of the remaining myocytes (21). While much of the developed literature have focused on myocyte hypertrophy, there is far less understanding about myocyte attrition, as a possible upstream phenomenon that occurs prior to adaptive hypertrophy. Cardiac muscle atrophy can occur in response to chronically reduced cardiac workload or to inflammatory disease states such as cancer (22). Interestingly, the failing heart can shrink and yet become stronger (23). In the studied cohorts of sarcopenic older patients with alterations in LVM, this phenomenon could be interpreted as pathologically adaptive with initial preservation of ejection fraction. Hence, there is value in targeting cardio-sarcopenia as an upstream phenotypic syndrome of concern in aging.

## Skeletal muscle sarcopenia and cardiac aging: Which one came first, or does it matter?

Recent literature has shown that patients with HF have worse muscle function and atrophy, because of pathological alterations including altered metabolism, energetics, and decreased oxidative capacity (24–27). Possible metabolic pathways linking physical activity, cardiovascular health, and musculoskeletal function with aging have also been described and summarized using insights from metabolomics (28). Of note, genes involved in fatty acid oxidation and glucose metabolism are upregulated by physical activity, whereas these changes are absent or reversed in HF (29). These differences in metabolic gene expression demonstrate that maladaptive cardiac hypertrophy elicited by pathological stimuli should be differentiated from adaptive exercise-induced hypertrophy.

Most of the literature that are involved with dysregulated skeletal muscles in clinical HF have provided us with some understanding of possibly shared pathways. It is therefore reasonable to suggest that a cross-talk may in fact pre-exist even before clinical CVD. Conjecturally, the cardio-sarcopenia syndrome likely results from a framework of partially shared pathways leading to the loss of functional mass involving more than one organ system, with a predilection for HF development. As there is a dearth of longitudinal studies tracking skeletal muscle mass and cardiac function prospectively, any suggestion of a causal link between the two remains speculative. Nevertheless, the co-occurrence of skeletal muscle sarcopenia and myocardial perturbations in older adults without and with co-morbidities observed by us and others may be consistent with common upstream pathological pathway (or pathways) associated with aging.

Recently, a chronic low-grade inflammatory state known as “inflammaging” has been described in older adults (30) that is characterized by elevations in blood inflammatory markers related to aging-related immune dysregulation, high circulating levels of proinflammatory markers such as interleukins, C-reactive protein (CRP), transforming growth factor-beta, tumor necrosis factor (TNF) and TNF receptors, including in those free of active disease (31–33). Observed in association with multi-systemic geriatric conditions, it has been postulated as a mechanism contributing to conditions ranging from sarcopenia to frailty and CVD (34–37). Interleukin (IL)-6 is a promising translational frailty biomarker in humans and mice (38). In a prospective, population-based study of 986 old men and women, high levels of serum IL-6 and CRP were associated with risks of muscle mass and strength loss (39). In another prospective cohort study of 620 old women, high serum IL-6 levels were associated with accelerated declines in muscle strength and physical function (40). IL-6 is a powerful independent predictor of HF (41, 42) and has been associated with impaired coronary flow and cardiac function, and worsening HF (43). Upregulated IL-6 activates gp130/STAT3 signaling, induces reactive oxygen species (ROS) production, leading to mitochondrial dysfunction and increased expression of mitophagy-related proteins, which results in cardiac hypertrophy in HF (44). Excessive ROS appears to aggravate ongoing inflammation, feeding a proinflammatory microenvironmental vicious cycle that exacerbates maladaptive myocardial remodeling and consequent HF manifestation (45, 46).

Insulin resistance features heavily in the biochemical cross-talk between the musculoskeletal and cardiovascular systems. Decreased mitochondrial function and increased inflammatory and oxidative stresses that are observed with skeletal muscle aging induce muscle atrophy and insulin resistance (47). The latter is central to an entity known as sarcopenic obesity (48), which is interestingly also associated with concentric LV remodeling independent of age-adjusted indexed body mass (49). From the metabolic perspective, it is plausible that insulin resistance mediates the cardiac remodeling associated with sarcopenic obesity, along with contributions by fat and inflammation. Further research chronicling the development of insulin resistance, sarcopenia, and cardiac remodeling (and HF) in aging human subjects is urgently needed to confirm this mechanistic link. If true, age-associated sarcopenia might be a pre-disease state that is amenable to upstream preventive strategies, e.g., exercise training, to avert clinical CVD.

Given the increased incidence of HF among older adults with increased risk of sarcopenia, these additional insights would support early preventative and/or therapeutic strategies that target specific aspects of skeletal muscles, potentially benefitting frail older adults who have yet to develop heart disease. For one, it highlights the potential of using screening tools such as SARC-F to identify older population with sarcopenia as an approach to detect early cardiac dysfunction in the community (50). The cardio-sarcopenic phenotype could also be targeted as a modifiable risk factor that may be ameliorated by interventions.

## Current detection tools

When considering the potential for scalability for case detection of older adults with cardio-sarcopenia syndrome in the geriatric population, detection tools should be easily accessible, safe with minimal radiation, non-invasive and robust. Currently, muscle mass can be estimated using bioelectrical impedance analysis (BIA) or dual-energy X-ray absorptiometry (DEXA) (5, 51), while myocardial structure and function can be assessed using echocardiography or cardiovascular magnetic resonance imaging, with newer variants including handheld echocardiography and the combined use of artificial intelligence tools (52, 53).

Importantly, some of these tools such as DEXA for body composition can be tagged onto existing imaging procedures for osteoporosis assessment of bone mineral density. Where available, the use of validated multi-frequency BIA facilitates access to measurement of muscle mass in the community setting, Lastly, as per the diagnostic algorithms of recent consensus statements for sarcopenia, the assessment of handgrip strength using a handheld dynamometer allows detection of “possible sarcopenia” defined by low muscle strength, circumventing the need for muscle mass measurement (54). Taken together, this allows clinicians to make more comprehensive risk assessment which combines inputs about the skeletal muscle in conjunction with more objective measurements of patients’ cardiac state (e.g., LVM).

## Potential to improve human health

Changing the focus from disease management to prevention is a paradigm shift that calls attention to the need for a systems approach to tackling cardiac aging and its complexities. While current HF guidelines recognizes the intimate link between sarcopenia and heart failure, more granular upstream preventative actions before the onset of clinical heart disease can potentially avert joint deteriorations in both organ systems. It is therefore timely to advocate stronger population-based preventative efforts for improving the health of older adults.

Exercise is important for maintaining and promoting skeletal and cardiac muscle health. Resistance training is effective for inducing skeletal muscle growth in older adults (55–57). In addition, there is growing evidence that aerobic exercise favorably affects various mechanisms that collectively stimulate skeletal muscle hypertrophy, and should therefore be considered as a viable exercise prescription in populations prone to muscle loss (58).

Dietary protein constitutes a primary nutrient for maintenance and growth of skeletal muscles (59). To combat sarcopenia, dietary guidelines recommend high protein intake for older adults, who are prone to low energy intake and muscle loss (60, 61). Individual nutrient effects on age-related skeletal muscle preservation (and cardioprotection) are less clear. To date, many studies on dietary fat and diverse micronutrients like whey, casein, Vitamin D, and antioxidants have yielded few definitive conclusions (62, 63).

Angiotensin-converting enzyme inhibitors (ACE-I) are routinely prescribed to patients with hypertension, HF and diabetes due to their beneficial effects on the vasculature and cardiovascular outcomes. At the molecular level, ACE-I promotes glucose uptake (64) and suppresses proinflammatory cytokines (especially IL-6) in skeletal muscles (65). In older adults without HF, ACE-I therapy has been shown to retard loss in muscle strength (66) and increase both muscle strength (67) and exercise capacity (68). Therefore, ACE-I or related pathways constitute promising therapeutic targets for sarcopenia.

On the other hand, sodium-glucose cotransporter-2 (SGLT2) inhibitors have recently been shown to confer renal and cardiometabolic benefits in diabetic and non-diabetic subjects (69–73) but may be associated with myopathy (74) and sarcopenia (75, 76) which will be of concern to aged adults with low baseline pre-treatment skeletal muscle mass. There is potential for SGLT2 inhibitors to be studied as a candidate drug targeting aging-related CVD, although the totality of clinical effectiveness for this class of therapeutics may depend on the needs of specific patient cohorts.

## Cardio-sarcopenia—Next steps

Firstly, there is a need for mechanistic understanding of the cross-talk between the various components of body composition and the cardiac muscles. Earlier research has revolved around obesity and its association with cardiovascular structure and function (77, 78). With emerging interest in skeletal muscles, sarcopenia enters the equation for the consideration of LVM. Notwithstanding the relative degree of involvement by adipose tissues or skeletal muscles, there is now greater awareness and scientific acknowledgment that there are close relationships between body composition and heart structure and function. This is in line with the opinion that the observed degenerative bodily changes in older adults is the result of the complex interplay between body composition, the cardiovascular system and aging process (79), far more granular than the body mass index metric (78).

In this regard, several studies have already demonstrated increased Framingham score or CVD risk in sarcopenic and/or obese older adults (80–82). In a large epidemiological study among the Korean population, it was observed that visceral obesity and low muscle mass may be patho-physiologically related, possibly through insulin resistance and inflammation, leading to subclinical LV changes independently and synergistically (83). Further elucidation of biological mechanisms that underlie these cardiometabolic alterations may require animal models.

Secondly, the cohort of older sarcopenic population should be followed up longitudinally with clear documentation of the changes in their skeletal and cardiac muscles as well as their cardiovascular status. This would provide clarity about the development of the cardio-sarcopenia syndrome in relation to the cardiac structure and function over time, which cannot be accomplished by cross-sectional studies that can only make associations at a single point in time. Concurrent biomarker annotation would provide additional mechanistic insights into the development of cardiac aging (84), useful for future clinical translation.

Thirdly, appropriate clinical trials should be conducted to evaluate the amount of change that is necessary to impact clinical outcomes. For example, in patients with clinically stable chronic HF (predominantly New York Heart Association class II or III), resistance exercise training can improve muscle strength (85, 86). More variability such as intensity and type of exercise should be trialed to determine the optimal level and type of physical exercise intervention for these patients. In addition to monitoring changes in the skeletal muscles, the cardiac structure and function can be concurrently assessed for any improvement over the course of the intervention. These features are not only therapeutic targets, but also markers requiring periodic surveillance, like blood pressure and low-density lipoprotein. The conduct of these interventional studies should additionally evaluate the impact of multi-component interventions such as nutrition, psychosocial support, and strategies personalized to individual needs (87).

## Conclusion

In conclusion, the concept of cardio-sarcopenia has evolved from a syndrome which describes the coexistence of alterations in myocardial structure with skeletal muscle sarcopenia in older adults to portend pathophysiologic derangements in the cardiac-skeletal muscle axis. Emerging evidence implicating the involvement of adipose tissue, such as sarcopenic obesity (88), highlights the need to examine deeper into the muscle-, fat- and myocardium triad. From the preventative standpoint, the cardio-sarcopenic phenotype constitutes a potentially modifiable risk factor in older persons that may be amenable to early multi-modal intervention involving physical activity, resistance exercise, and nutrition. This raises the clarion call for greater inter-disciplinary collaboration between cardiologists, geriatricians, bio-scientists, exercise therapists, and nutrition specialists to push the frontiers in both research and clinical practice against this syndrome of concern in aging.

## Author contributions

DRL, WSL, and ASK contributed to the conception and design of the manuscript. RST, DRL, WSL, and ASK performed literature critiques and contributed to the writing of the manuscript. All authors critically reviewed previous drafts and approved the final draft for submission.
